# Surgical options for Evans-Jensen type IV intertrochanteric femur fractures in the elderly over 65: a comparison between total hip arthroplasty and proximal femoral nail antirotation

**DOI:** 10.3389/fsurg.2024.1510094

**Published:** 2025-01-07

**Authors:** Ming Sun, Hai-Rui Liang, He Zhang, Tong Bai, Rong-Da Xu, Si-Yu Duan, Zhen-Cun Cai

**Affiliations:** ^1^ Department of Orthopedics Surgery, Central Hospital Affiliated to Shenyang Medical College, Shenyang, Liaoning, China; ^2^ Sports Medicine, Tongliao People’s Hospital, Tongliao, Inner Mongolia, China; ^3^ Department of Orthopedics Surgery, Shenyang Ninth People’s Hospital, Shenyang, Liaoning, China; ^4^ Liaoning Province Key Laboratory for Phenomics of Human Ethnic Specificity and Critical Illness, and Shenyang Key Laboratory for Phenomics, Shenyang, Liaoning, China

**Keywords:** intertrochanteric fracture, proximal femoral nail antirotation, total hip arthroplasty, Evans-Jensen type IV, geriatric medicine

## Abstract

**Background:**

Currently, there is no clear standard for the surgical options for Evans-Jensen Type IV intertrochanteric femur fractures in elderly patients over 65 years old. This study aims to retrospectively analyze and compare the early postoperative limb function and quality of life of patients treated with total hip arthroplasty (THA) and proximal femoral nail antirotation (PFNA) for this type of fracture. We hypothesize that there is no significant difference in complications and postoperative recovery between the two surgical methods.

**Methods:**

A retrospective analysis was conducted on patients over 65 years old with Evans-Jensen Type IV intertrochanteric femur fractures who were treated between 2020 and 2023. The patients were divided into two groups based on the treatment method: the PFNA group (20 cases) and the THA group (20 cases). General patient information, operative time, intraoperative blood loss, time to postoperative mobilization, time to weight-bearing on the affected limb, Harris hip scores at 1, 3, and 6 months postoperatively, excellent and good rates, SF-36 scores, and postoperative complications were recorded.

**Results:**

Compared to the PFNA group, the THA group had a longer operative time (86.7 ± 9.6 vs. 51.5 ± 5.3 min, *p* < 0.001) and more intraoperative blood loss (212.0 ± 35.5 vs. 76.5 ± 16.1 ml, *p* < 0.001). However, the THA group had an earlier time to first postoperative mobilization (3.1 ± 1.4 vs. 43.3 ± 13.09 days, *p* < 0.001) and earlier time to full weight-bearing on the affected limb (33.5 ± 3.1 vs. 77.9 ± 12.0 days, *p* < 0.001). The Harris hip scores and SF-36 scores at 1, 3, and 6 months postoperatively were higher in the THA group (*p* < 0.05 for all). There was no significant difference in the overall incidence of postoperative complications between the two groups (*p* = 0.41).

**Conclusion:**

For elderly patients over 65 years old with Evans-Jensen Type IV intertrochanteric femur fractures, especially those with underlying diseases who cannot tolerate prolonged bed rest, hip replacement surgery (THA) may be preferred. Postoperative patients can begin rehabilitation exercises earlier, preventing the exacerbation of internal medical conditions. Early recovery of hip function on the affected side is faster, and the quality of life of patients is higher.

## Introduction

Intertrochanteric Fracture (ITF) commonly occurs in elderly patients, with a high incidence rate of approximately 3.4% (1). In elderly patients, these fractures are often comminuted and unstable, and are frequently accompanied by osteoporosis, hypertension, diabetes, and cardiovascular diseases, resulting in poor physical function. With the aging global population, the incidence of unstable intertrochanteric fractures is expected to increase annually (2). The goal of surgical treatment for elderly patients with intertrochanteric fractures is to restore the hip structure, stabilize the fracture, and allow early mobilization, thereby preventing complications such as bedsores, infections, and deep vein thrombosis of the lower limbs caused by prolonged bed rest, as well as preventing the exacerbation of underlying diseases and reducing mortality (3).

Regarding the treatment of Type IV ITF, some scholars advocate the use of Proximal Femoral Nail Antirotation (PFNA) (4, 5). However, in elderly patients, PFNA is associated with a high incidence of complications such as fracture displacement, femoral neck screw cut-out, and hip varus deformity postoperatively (6, 7). Consequently, some scholars propose total hip arthroplasty (THA) as an alternative, as it can reconstruct the hip structure and allow for early mobilization, avoiding complications associated with PFNA. Currently, there is still controversy regarding the optimal surgical approach for patients over 65 years old with Evans-Jensen Type IV intertrochanteric fractures (8).

Through a literature review, we found that there is no specific study focusing on the best surgical method for this particular patient group. This study aims to retrospectively compare the differences between the two surgical approaches in terms of perioperative outcomes, hip function, quality of life, and postoperative complications in patients over 65 with Evans-Jensen Type IV ITF, providing a reference for the selection of surgical methods in clinical practice for elderly patients with Evans-Jensen Type IV ITF.

## Methods

A retrospective analysis was conducted on 40 patients aged 65 and older with intertrochanteric fractures (ITF) admitted to the Affiliated Central Hospital of Shenyang Medical College between 2020 and 2023. A total of 20 patients who underwent hip replacement surgery were included in the THA group. Another 20 patients with the same fracture type who were treated with PFNA served as the control group, defined as the PFNA group. All patients were informed of the differences between PFNA and THA treatment options and chose the surgical method based on their personal preferences.

### Inclusion criteria

Patients over 65 years old with newly diagnosed closed fractures, Evans-Jensen Type IV fractures, and who met the surgical indications for PFNA or THA.

### Exclusion criteria

Patients with pathological fractures, those unable to undergo surgery due to severe medical conditions, patients who were lost to follow-up, and patients whose postoperative limb function assessment was affected by other diseases.

### Ethical approval

This study was approved by the Medical Ethics Committee of the Affiliated Central Hospital of Shenyang Medical College, and informed consent was obtained from all patients and their families.

#### Preoperative patient preparation and management

After admission, patients received skin traction on the affected limb, swelling reduction, pain relief, and treatment to prevent deep vein thrombosis of the lower limbs. Preoperative examinations were completed. All patients underwent preoperative hip x-rays (Philips Digital Radiography System, Netherlands) and CT scans of the affected hip with three-dimensional reconstruction (Philips 256-slice spiral CT scanner, Netherlands, with a slice thickness of 0.6 mm).

#### Surgical methods and brief procedure

##### Anesthesia

The anesthesia method used was either spinal anesthesia or general anesthesia.

##### PFNA group

The patient was placed in a supine position, with the affected limb fixed on the traction table. Under fluoroscopy, a closed reduction of the fracture ends was performed. A longitudinal incision was made approximately 3 cm above the greater trochanter, followed by layer-by-layer dissection to expose the greater trochanter. The main femoral nail, femoral neck helical screws, and distal locking screws were inserted under fluoroscopy, paying attention to the anteversion angle and tip distance. Finally, the proximal intramedullary nail cap was screwed in. Muscle contraction exercises began the day after surgery, and monthly follow-up hip x-rays were performed to determine the time for mobilization and weight-bearing based on fracture healing.

##### THA group

**Figure 1:**
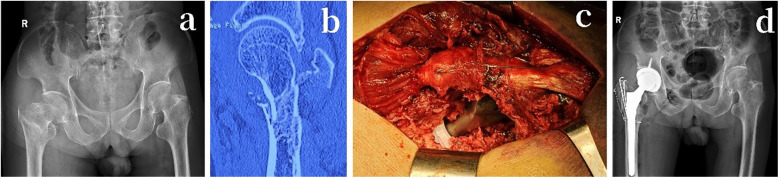
An 78-year-old male patient with a right-sided Evans-Jensen type IV intertrochanteric femur fracture due to a fall. **(a)** Preoperative anteroposterior x-ray of both hip joints. **(b)** Preoperative coronal CT scan. **(c)** Intraoperative total hip arthroplasty, with the use of Kirschner wires for tension band fixation of the greater trochanter and titanium cables for securing the proximal femur. **(d)** Postoperative anteroposterior x-ray of both hip joints on the first day after surgery.

The patient was positioned on the side of the unaffected limb, and a lateral approach to the hip joint was used. Part of the external rotator muscle group was cut at the insertion point of the greater trochanter, while preserving the piriformis muscle and as much of the medial femoral distance as possible. A suitably sized acetabular cup and liner were installed, taking care to ensure proper abduction and anteversion angles. The lesser trochanter fracture was reduced and fixed, and a titanium cable was used to bundle the proximal femur to prevent fracture during the insertion of the femoral prosthesis. A properly sized Wagner femoral stem was implanted, ensuring the anteversion angle was correct and that both lower limbs were of equal length. The greater trochanter fracture fragments were reduced and fixed with a tension band. Postoperatively, functional exercises were initiated based on the patient's condition, with the gradual introduction of weight-bearing practice using assistive devices as needed (Figure 1).

#### Postoperative management

Postoperatively, active treatment of underlying diseases continued, along with infection prevention and prevention of deep vein thrombosis in the lower limbs. Preoperative x-rays (Figure 1a), preoperative CT scans (Figure 1b), and postoperative x-rays taken on the first day (Figure 1d) were retained to compare and evaluate the surgical outcomes.

After the patient recovered from anesthesia, they were guided to perform ankle pump exercises, along with contraction and relaxation exercises for the knee and hip muscles. Patients in the hip replacement group were instructed to begin walking exercises with the assistance of a walker as early as possible. In the PFNA group, patients were advised to perform appropriate ankle pump and muscle contraction exercises while in bed. Follow-up x-rays of both hips were performed at 1, 2, and 3 months to assess hip recovery and fracture healing, and to guide patients on postoperative rehabilitation exercises and full weight-bearing activities. x-rays of both hips may be reviewed at 6 and 12 months postoperatively as needed to evaluate the recovery status of the fracture.

##### Clinical efficacy evaluation indicators

*Follow-up was conducted primarily through telephone and outpatient visits at 1 month, 3 months, and 6 months postoperatively. During the follow-up, patient scoring surveys were conducted, imaging examinations were completed, and relevant data were collected*.

1. *Recording surgical and rehabilitation data:*
  *Surgical duration: Time from skin incision to skin closure;*
  *Intraoperative blood loss: Volume of blood collected in the suction device and gauze;*
  *Time to first postoperative ambulation (T1) and time to full weight-bearing on the affected limb (T2).*
2. *Functional assessment:*
  *Postoperative Harris hip scores and excellent rates for the affected hip were evaluated at 1 month, 3 months, and 6 months for both groups;*
  *Excellent: 90–100 points, Good: 80–89 points, Fair: 70–79 points, Poor: below 70 points* (9)*.*
3. *SF-36 Quality of Life Questionnaire* (10):
  *The SF-36 consists of 36 items and is a brief survey designed to assess health and functional status across various age groups, different diseases, and control populations.*
  *It includes 8 dimensions: physical functioning, role physical, bodily pain, general health, social functioning, role emotional, and mental health. The total score across these dimensions represents the overall score of the questionnaire.*
4. *Postoperative complications:*
  *Includes infections, internal fixation failure, hip dislocation, etc.*

#### Statistical analysis

All analyses were conducted using SPSS 26.0 software (IBM, Armonk, NY, USA). Categorical variables were presented as numbers or percentages. Continuous variables were expressed as means ± standard deviation. Categorical variables were evaluated using Fisher's exact test. The Shapiro-Wilk test was used to determine whether continuous variables followed a normal distribution; continuous variables that were normally distributed were analyzed using independent samples *t*-tests. The significance level for all statistical tests was set at *p* < 0.05.

## Results

### Baseline and surgical data, including age, sex, surgical time, and intraoperative blood loss, were collected

A total of 40 patients with Type IV ITF were included, with 20 patients in the PFNA group (4 males, 16 females) and an average age of 72.8 ± 5.2 years. The THA group also had 20 patients (6 males, 14 females) with an average age of 73.7 ± 5.5 years. There were no statistically significant differences in age or sex between the two groups (*p* > 0.05) (Table 1).

**Table 1:** Baseline and surgical data.

Characteristics	PFNA (*n* = 20)	THA (*n* = 20)	*p*
Age (years)	72.8 ± 5.2	73.7 ± 5.5	0.27
Gender, *n*			0.72
Male	4	6	
Female	16	14	
BMI, kg/m^2^	24.4 ± 3.0	24.9 ± 3.2	0.66
Surgery time (minutes)	51.5 ± 5.3	86.7 ± 9.6	<0.001
Intraoperative bleeding volume (ml)	76.5 ± 16.1	212.0 ± 35.5	<0.001
Hemoglobin, g/L	89.7 ± 16.8	90.8 ± 17.4	0.83
Liver function abnormalities, *n*			0.73
Abnormal	5	7	
Normal	15	13	
Kidney function abnormalities, *n*			0.72
Abnormal	6	4	
Normal	14	16	
Electrocardiogram (ECG) abnormalities, *n*			0.70
Abnormal	3	5	
Normal	17	15	
Lung function abnormalities, *n*			0.41
Abnormal	5	2	
Normal	15	18	

The PFNA group had a shorter surgical time (51.5 ± 5.3 vs. 86.7 ± 9.6 min, *p* < 0.001), and the intraoperative blood loss was less in the PFNA group (76.5 ± 16.1 vs. 212.0 ± 35.5 ml, *p* < 0.001). The differences were statistically significant (Table 1).

### Functional scores and prognosis assessment

The THA group demonstrated an earlier time to first mobilization (3.1 ± 1.4 vs. 43.3 ± 13.09 days, *p* < 0.001) and an earlier time to full weight-bearing on the affected limb (33.5 ± 3.1 vs. 77.9 ± 12.0 days, *p* < 0.001). These differences were statistically significant.

In follow-up evaluations at 1, 3, and 6 months postoperatively, the Harris scores showed that the excellent/good rate in the PFNA group was 10%, 55%, and 55%, respectively, while the THA group had rates of 70%, 85%, and 90%. The hip function scores in the THA group were significantly higher than those in the PFNA group (1st month *p* < 0.001, 3rd month *p* = 0.04, 6th month *p* = 0.02).

For the SF-36 scale, the THA group scored higher than the PFNA group at all time points: 1 month (75.1 ± 4.0 vs. 66.1 ± 8.0, *p* < 0.001), 3 months (82.3 ± 3.7 vs. 79.4 ± 4.8, *p* = 0.04), and 6 months (88.7 ± 2.8 vs. 85.5 ± 2.6, *p* = 0.001). All differences were statistically significant (Table 2).

**Table 2:** Functional scores and prognostic indicators.

Characteristics	PFNA (*n* = 20)	THA (*n* = 20)	*p*
First weight-bearing time, days	43.3 ± 13.0	3.1 ± 1.4	<0.001
Full weight-bearing time, days	77.9 ± 12.0	33.5 ± 3.1	<0.001
Harris score, *n* (%)			
First month excellent/good (≥80 points)	2 (10)	14 (70)	<0.001
Fair/poor (<80 points)	18 (90)	6 (30)	
Third month excellent/good (≥80 points)	11 (55)	17 (85)	0.04
Fair/poor (<80 points)	9 (45)	3 (15)	
Sixth month excellent/good (≥80 points)	11 (55)	18 (90)	0.02
Fair/poor (<80 points)	9 (45)	2 (10)	
SF-36 scale, scores			
First month	66.1 ± 8.0	75.1 ± 4.0	<0.001
Third month	79.4 ± 4.8	82.3 ± 3.7	0.04
Sixth month	85.5 ± 2.6	88.7 ± 2.8	0.001

**Table 3:** Postoperative complications statistics for two groups of patients (*n* = 20).

Group	Overall complications	Specific complications			
		Infection	Screw cutting	Hip joint dislocation	Unequal leg length
PFNA	5	2	3	0	0
THA	2	0	0	1	1
p	0.41				

### Postoperative complications

**Figure 2:**
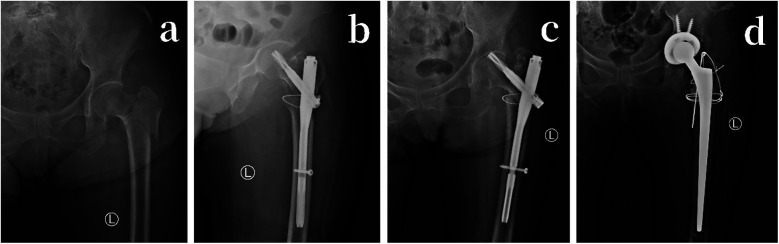
**(a)** 73-year-old female with a fall-induced intertrochanteric fracture of the left femur. **(a)** Preoperative anteroposterior x-ray of the left hip joint. **(b)** Postoperative first-day anteroposterior x-ray of the left hip joint, using PFNA internal fixation with external lateral wall bundling with titanium cables. **(c)** Postoperative 5-month anteroposterior x-ray of the left hip joint, showing proximal screw cutting of PFNA. **(d)** Removal of PFNA internal fixation, followed by total hip joint replacement surgery; postoperative first-day anteroposterior x-ray of the left hip joint.

The PFNA group had more postoperative complications, with a total of 5 cases, including: 2 cases of pulmonary infection, both cured after anti-infective treatment in the respiratory department. 2 cases of screw cut-out at the femoral head, both of which required a second-stage total hip replacement (Figure 2). 1 case of screw cut-out at the femoral neck, considered to be due to improper screw placement, which was treated with revision surgery and repositioning of the screw.

In the THA group, there were 2 postoperative complications: 1 case of hip dislocation, which occurred in a patient with cerebral atrophy due to excessive hip flexion postoperatively. This was treated with closed reduction.1 case of limb length discrepancy, where the affected limb was 1.6 cm longer than the healthy side. Since there was no significant limping, no further intervention was required (Table 3).

## Discussion

Elderly patients with intertrochanteric fractures (ITF) who have uncontrolled underlying medical conditions generally have poor overall health, making them unable to tolerate anesthesia and surgery, thus necessitating conservative treatment. However, the mortality rate for conservative treatment within one year can be as high as 84.4% (11). With advancements in medical technology and the introduction and application of Enhanced Recovery After Surgery (ERAS) principles (12), surgical treatment opportunities for elderly ITF patients have significantly increased, and surgery has become the primary treatment method for this injury (13).

Intramedullary fixation and total hip arthroplasty (THA) are currently the mainstream surgical methods for treating type IV ITF. However, there are several contentious points (14–17): Performing PFNA on unstable fractures can present challenges with preoperative reduction, and it is often difficult to effectively stabilize fragmented bone during surgery. This results in a high incidence of postoperative internal fixation failure (18, 19), which adversely affects fracture healing, and revision surgeries can cause additional trauma (20). Patients with associated lateral wall deficiencies often require additional internal fixation treatments, such as greater trochanteric plating. THA carries the risk of intraoperative vascular and sciatic nerve injuries, significant blood loss, and severe damage to the surrounding structures of the hip. There is also a risk of femoral fracture during the procedure, and if bone cement is used during surgery, there is a possibility of cement-related complications (21). Furthermore, there is ongoing debate regarding the choice of prosthesis type during the surgery.

In this study, the SF-36 scores, Harris scores, and excellent rates at each follow-up stage for the THA group were all higher than those for the PFNA group. Possible reasons for this include: THA can prevent discomfort caused by the wear of the metal femoral head against the bony structure of the acetabulum; the surgery preserves the piriformis and part of the external rotator muscles, which is crucial as patients often present with greater trochanter fractures and displacements, thus increasing the likelihood of successfully retaining these external rotator muscles; intraoperatively suturing and repairing torn or incised joint capsules effectively reduces trauma, restores hip structure, and lowers the risk of postoperative hip dislocation (22). Additionally, using titanium cables to bundle the proximal femur before inserting the femoral prosthesis can prevent femoral fracture; employing titanium cables to stabilize the lesser trochanter fracture and using K-wires and steel wires in a tension band configuration to stabilize the greater trochanter fracture effectively restores and stabilizes the fractures, restoring the hip musculature and bony structure.

The use of the Wagner SL biological femoral stem in this study offers several advantages (23): the stability of the SL stem relies on the mid- to distal femur, providing a stable biomechanical structure for the hip postoperatively; the anteversion angle can be freely adjusted during the procedure to prevent postoperative hip dislocation; and the femoral side is shaped by drilling into the medullary cavity, which avoids stress concentration and reduces the risk of femoral fracture. In the PFNA group, the internal fixation method only involved three screws of varying lengths, while the proximal structure in IV-type ITF is compromised, leading to instability in the fixation of both the greater and lesser trochanters. During postoperative activities, the iliopsoas and gluteal muscles can exert strong traction on the fractured fragments, resulting in significant pain; early weight-bearing can also increase the risk of internal fixation failure and the occurrence of hip internal rotation (especially in type IV fractures). Therefore, clinicians often determine the timing for full weight-bearing based on the healing progress of the fracture in PFNA patients. Consequently, the THA group achieved early rehabilitation and weight-bearing, which is beneficial for the recovery of cardiopulmonary function and the function of the affected hip joint.

*Our research team believes that for patients over 65 years old with multiple underlying diseases and severe osteoporosis suffering from Evans-Jensen type IV intertrochanteric fractures, Wagner SL biological stem total hip arthroplasty (THA) is the recommended first-choice treatment. Intraoperative injection of a* “*cocktail mix of analgesics*” *significantly reduces early postoperative pain* (24)*. Additionally, the preoperative and intraoperative use of tranexamic acid* (25) *effectively reduces intraoperative blood loss, avoiding the need for blood transfusion*.

*Although total hip arthroplasty involves significant surgical trauma, may lead to postoperative limb length discrepancies, and has limitations regarding the lifespan of the prosthesis, considering the patients' age and health status, THA remains the superior choice. For patients with Evans-Jensen type IV intertrochanteric fractures, while proximal femoral nail anti-rotation (PFNA) treatment aligns with modern minimally invasive surgical principles, it carries a high risk of screw cut-out (incidence rate of 15%, i.e., 3 out of 20 cases), which may necessitate secondary surgery. Moreover, due to the requirement for postoperative bed rest, factors such as pain and infection increase the likelihood of cardiopulmonary function deterioration. For elderly patients, these issues can further impact survival rates. Therefore, we recommend prioritizing total hip arthroplasty (THA) for this specific patient population to reduce the incidence of secondary surgeries, lower the risk of postoperative complications, promote early rehabilitation and recovery, improve patients' quality of life, and ultimately enhance survival rates. Although extramedullary fixation and conservative treatment are alternative options, we do not recommend them as first-line treatments for elderly patients with intertrochanteric fractures*.

*This study has certain limitations. Firstly, it is a retrospective and non-randomized study. Secondly, all surgeries were performed by the same research team, and the clinical outcomes may vary due to differences in the surgeons' individual experience. Future studies could consider incorporating computer-assisted robotic surgery to address this limitation. Lastly, this research represents an initial study, and the relatively small sample size may affect the accuracy of the experimental results. Further multi-center, large-sample, and long-term follow-up studies are needed to validate our conclusions*.

## Conclusion

For patients aged 65 and older with Evans-Jensen IV-type intertrochanteric fractures, especially those with comorbidities who cannot tolerate prolonged bed rest, hip arthroplasty should be considered as a treatment option. This approach allows patients to engage in rehabilitation exercises earlier after surgery, prevents the exacerbation of underlying medical conditions, and facilitates faster recovery of hip function on the affected side, leading to improved quality of life for the patients.

## Data Availability

The original contributions presented in the study are included in the article/Supplementary Material, further inquiries can be directed to the corresponding author.
